# Supplementary material for: Depressive symptoms and other risk factors predicting suicide in middle-aged men: a prospective cohort study among Korean Vietnam War veterans
Source: PeerJ. 2015 Jul 2;3:e1071. doi: 10.7717/peerj.1071 (PMC4493683; doi:10.7717/peerj.1071)
Supplement: Table S2 — BDI, Beck Depression Inventory; CI, confidence interval; HR, hazard ratio. a. Hazard ratios were calculated using a Cox proportional hazards model over multiple imputed data, after adjustment for age at enrollment, smoking status, drinking status, body mass index, self-rated health, marital status, educational status, household monthly income and participant group. b. Number of suicides may not match those of the other groups of depressive symptoms due to rounding of averages over multiple imputed data. c. Cut-off score based on previous research. d. Five categories of depressive symptoms were analyzed as ordinal variables. [file peerj-03-1071-s002.pdf]

Table S2. Adjusted hazard ratios of suicides by depressive symptoms according to follow-up period[a] (Sensitivity analysis)

Categories of depressive symptoms (score)		From 2001 to 2004 n=10,238			From 2005 to 2008 n=9,877		
		No. of suicides[b]	p-value	HR (95% CI)	No. of suicides[b]	p-value	HR (95% CI)
2-group analysis (2)[c]	No-to-moderate (0-29)	4		1.0 [Reference]	10		1.0 [Reference]
	Severe (30-63)	12	0.03	4.3 (1.1-16.5)	15	0.09	2.6 (0.9-7.6)
5-group analysis (2)[c]	No (0-9)	0		1.0 [Reference]	1		1.0 [Reference]
	Mild (10-16)	3		1.0 [Reference]	3	0.87	1.2 (0.1-14.5)
	Moderate (17-29)	1		1.0 [Reference]	5	0.76	1.5 (0.1-17.2)
	Severe (30-40)	5	0.12	3.3 (0.7-14.9)	9	0.33	3.3 (0.3-37.8)
	Extreme (41-63)	7	0.02	7.6 (1.5-39.5)	7	0.26	4.3 (0.3-54.2)
	Trend test[d]	16	0.04	2.0 (1.0-3.8)	25	0.08	1.5 (0.9-2.5)

BDI, Beck Depression Inventory; CI, confidence interval; HR, hazard ratio.

a. Hazard ratios were calculated using a Cox proportional hazards model over multiple imputed data, after adjustment for age at enrollment, smoking status, drinking status, body mass index, self-rated health, marital status, educational status, household monthly income and participant group.

b. Number of suicides may not match those of the other groups of depressive symptoms due to rounding of averages over multiple imputed data.

c. Cut-off score based on previous research

d. Five categories of depressive symptoms were analyzed as ordinal variables
