# Supplementary material for: Depressive symptoms and other risk factors predicting suicide in middle-aged men: a prospective cohort study among Korean Vietnam War veterans
Source: PeerJ. 2015 Jul 2;3:e1071. doi: 10.7717/peerj.1071 (PMC4493683; doi:10.7717/peerj.1071)
Supplement: Table S3 — BDI, Beck Depression Inventory; CI, confidence interval; HR, hazard ratio. a. Hazard ratios were calculated using a Cox proportional hazards model over multiple imputed data, after adjustment for age at enrollment, smoking status, drinking status, body mass index, self-rated health, marital status, educational status, household monthly income, participant group and depressive symptoms (5 categories based on quartiles and the last decile of the total BDI score). b. Sum of the number of suicides by each risk factor may not equal the total suicides due to rounding of averages over multiple imputed data. c. Categories of risk factors were analyzed as ordinal variables. d. 1,300 Korean Won was about one US dollar as of June, 2001. [file peerj-03-1071-s003.pdf]

Table S3. Adjusted hazard ratios of suicides by risk factors according to follow-up period[a]

Risk factors	Categories	From 2001 to 2004 n=10,238			From 2005 to 2008 n=9,877		
		No. of suicides[b]	p-value	HR (95% CI)	No. of suicides[b]	p-value	HR (95% CI)
Age at enrollment	One year increase in age	16	0.23	0.9 (0.7-1.1)	25	0.65	1.0 (0.8-1.1)
Smoking status	Never smoker	2		1.0 [Reference]	7		1.0 [Reference]
	Past smoker	4	0.6	1.6 (0.3-8.7)	3	0.23	0.4 (0.1-1.7)
	Current smoker	10	0.3	2.3 (0.5-10.5)	15	0.92	1.0 (0.4-2.7)
	p for trend[c]	16	0.27	1.5 (0.7-3)	25	0.79	1.1 (0.6-1.8)
Drinking status	Never drinker	0		1.0 [Reference]	1		1.0 [Reference]
	Past drinker	8	0.13	26.3 (0.4-1787.6)	8	0.2	5.7 (0.4-82.1)
	Current drinker	8	0.22	14.1 (0.2-944.3)	15	0.24	4.9 (0.3-69.2)
	p for trend[c]	16	0.53	1.3 (0.6-2.7)	25	0.37	1.3 (0.7-2.6)
Body mass index	Below 18.5	0	0.99	0.0	2	0.47	1.8 (0.4-8.1)
(kg/m^2)	18.5-22.9	11		1.0 [Reference]	11		1.0 [Reference]
	23.0-24.9	4	0.48	0.7 (0.2-2.1)	6	0.9	0.9 (0.3-2.6)
	25 or above	1	0.1	0.2 (0-1.4)	6	0.99	1.0 (0.4-2.7)
	p for trend[c]	16	0.22	0.7 (0.4-1.3)	25	0.73	0.9 (0.6-1.5)
Self-rated health	Very good or good	0		0.0	1	0.77	1.4 (0.1-14.6)
	Fair	4	0.32	2.1 (0.5-9.4)	5	0.83	0.9 (0.3-2.8)
	Poor	5		1.0 [Reference]	11		1.0 [Reference]
	Very poor	7	0.08	3.0 (0.9-10.3)	8	0.13	2.1 (0.8-5.7)
	p for trend[c]	16	0.33	1.5 (0.7-3.5)	25	0.26	1.5 (0.8-2.8)

Risk factors	Categories	From 2001 to 2004 n=10,238			From 2005 to 2008 n=9,877		
		No. of suicides[b]	p-value	HR (95% CI)	No. of suicides[b]	p-value	HR (95% CI)
Marital status	Living with spouse	14		1.0 [Reference]	21		1.0 [Reference]
	Living without spouse	2	0.61	0.7 (0.1-3.1)	4	0.45	1.5 (0.5-4.7)
Educational status	Elementary school or below	4	0.82	1.2 (0.3-5.0)	12	0.02	3.5 (1.2-10.2)
	Middle school	8	0.15	2.5 (0.7-8.4)	7	0.36	1.8 (0.5-5.8)
	High school or above	4		1.0 [Reference]	6		1.0 [Reference]
	p for trend[c]	16	0.56	0.8 (0.5-1.5)	25	0.02	0.6 (0.3-0.9)
Household income	Below 1 000 000	10	0.59	1.9 (0.2-17.6)	11	0.97	1.0 (0.2-5.1)
per month	1 000 000-1 990 000	5	0.89	1.2 (0.1-11.2)	11	0.81	1.2 (0.2-5.9)
(Korean Won)[d]	2 000 000 or more	1		1.0 [Reference]	3		1.0 [Reference]
	p for trend[c]	16	0.45	0.7 (0.3-1.8)	25	0.81	1.1 (0.5-2.3)
Participant group	Health exam group	3	0.69	1.3 (0.4-4.6)	4	0.84	0.9 (0.3-2.6)
	Survey-only group	13		1.0 [Reference]	21		1.0 [Reference]

BDI, Beck Depression Inventory; CI, confidence interval; HR, hazard ratio.

a. Hazard ratios were calculated using a Cox proportional hazards model over multiple imputed data, after adjustment for age at enrollment, smoking status, drinking status, body mass index, self-rated health, marital status, educational status, household monthly income, participant group and depressive symptoms (5 categories based on quartiles and the last decile of the total BDI score).

b. Sum of the number of suicides by each risk factor may not equal the total suicides due to rounding of averages over multiple imputed data.

c. Categories of risk factors were analyzed as ordinal variables.

d. 1300 Korean Won was about one US dollar as of June, 2001
