# Supplementary material for: Depressive symptoms and other risk factors predicting suicide in middle-aged men: a prospective cohort study among Korean Vietnam War veterans
Source: PeerJ. 2015 Jul 2;3:e1071. doi: 10.7717/peerj.1071 (PMC4493683; doi:10.7717/peerj.1071)
Supplement: Table S4 — AUC, area under the receiver operating characteristics curve; CI, confidence interval. a. Cut-off score based on previous research. b. 95% CI was calculated using Wilson score method. c. Proportion of true positives (suicides) correctly identified. d. Proportion of true negatives (non-suicides) correctly identified. e. A diagnostic test with an AUC value of 1.0 or 0.5 represents a perfect test or an uninformative test, respectively. [file peerj-03-1071-s004.pdf]

**Table S4. Diagnostic characteristics of Beck Depression Inventory for suicide by follow-up period in Korean middle-aged men.** **(Sensitivity analysis)**

| | | Cut-off of 30 or above[a] | Unbinned Total Score |
|---|---|---|---|
| **Follow-up period** | **Characteristics** | **Rate (95% CI)[b]** | **Rate (95% CI)[b]** |
| From 2001-2008 | Sensitivity[c] | 67 (52-79) | |
| | Specificity[d] | 71 (70-72) | |
| | AUC[e] | 0.69 (0.61-0.76) | 0.71 (0.63-0.80) |
| From 2001-2004 | Sensitivity[c] | 75 (50-90) | |
| | Specificity[d] | 71 (70-71) | |
| | AUC[e] | 0.73 (0.62-0.84) | 0.75 (0.61-0.90) |
| From 2005-2008 | Sensitivity[c] | 62 (42-78) | |
| | Specificity[d] | 71 (71-72) | |
| | AUC[e] | 0.67 (0.57-0.76) | 0.69 (0.58-0.80) |

AUC, area under the receiver operating characteristics curve; CI, confidence interval.
a. Cut-off score based on previous research
b. 95% CI was calculated using Wilson score method
c. Proportion of true positives (suicides) correctly identified
d. Proportion of true negatives (non-suicides) correctly identified
e. A diagnostic test with an AUC value of 1.0 or 0.5 represents a perfect test or an uninformative test, respectively
