# Supplementary material for: Depressive symptoms and other risk factors predicting suicide in middle-aged men: a prospective cohort study among Korean Vietnam War veterans
Source: PeerJ. 2015 Jul 2;3:e1071. doi: 10.7717/peerj.1071 (PMC4493683; doi:10.7717/peerj.1071)
Supplement: Table S5 — AUC, area under the receiver operating characteristics curve; BDI, Beck Depression Inventory; CI, confidence interval. a. A diagnostic test with an AUC value of 1.0 or 0.5 represents a perfect test or an uninformative test, respectively. [file peerj-03-1071-s005.pdf]

Table S5. AUC for suicide by cut-off score of Beck Depression Inventory in Korean middle-aged men.

BDI score cut-off	AUC[a] (95% CI)	BDI score cut-off	AUC[a] (95% CI)
5 or above	0.510 (0.486-0.535)	31 or above	0.694 (0.620-0.767)
6 or above	0.517 (0.492-0.541)	32 or above	0.686 (0.611-0.762)
7 or above	0.523 (0.498-0.548)	33 or above	0.652 (0.574-0.729)
8 or above	0.531 (0.506-0.557)	34 or above	0.656 (0.578-0.733)
9 or above	0.541 (0.516-0.566)	35 or above	0.663 (0.585-0.740)
10 or above	0.551 (0.526-0.577)	36 or above	0.657 (0.580-0.735)
11 or above	0.550 (0.515-0.584)	37 or above	0.663 (0.586-0.741)
12 or above	0.550 (0.509-0.592)	38 or above	0.644 (0.567-0.720)
13 or above	0.563 (0.521-0.605)	39 or above	0.648 (0.572-0.724)
14 or above	0.576 (0.533-0.619)	40 or above	0.623 (0.549-0.697)
15 or above	0.577 (0.528-0.625)	41 or above	0.619 (0.546-0.692)
16 or above	0.567 (0.509-0.624)	42 or above	0.617 (0.545-0.689)
17 or above	0.580 (0.522-0.638)	43 or above	0.603 (0.534-0.673)
18 or above	0.592 (0.533-0.651)	44 or above	0.578 (0.514-0.643)
19 or above	0.604 (0.544-0.664)	45 or above	0.577 (0.514-0.641)
20 or above	0.617 (0.557-0.678)	46 or above	0.576 (0.513-0.638)
21 or above	0.617 (0.553-0.682)	47 or above	0.562 (0.504-0.620)
22 or above	0.629 (0.564-0.694)	48 or above	0.558 (0.502-0.614)
23 or above	0.641 (0.575-0.707)	49 or above	0.539 (0.490-0.587)
24 or above	0.653 (0.587-0.720)	50 or above	0.532 (0.488-0.576)
25 or above	0.667 (0.600-0.734)	51 or above	0.531 (0.488-0.573)
26 or above	0.678 (0.611-0.746)	52 or above	0.530 (0.488-0.571)
27 or above	0.664 (0.592-0.736)	53 or above	0.516 (0.484-0.549)
28 or above	0.675 (0.603-0.747)	54 or above	0.511 (0.483-0.538)
29 or above	0.682 (0.610-0.755)	55 or above	0.509 (0.484-0.533)
30 or above	0.686 (0.612-0.759)	56 or above	0.509 (0.485-0.533)

AUC, area under the receiver operating characteristics curve; BDI, Beck Depression Inventory; CI, confidence interval.

a. A diagnostic test with an AUC value of 1.0 or 0.5 represents a perfect test or an uninformative test, respectively
