# Supplementary material for: Depressive symptoms and other risk factors predicting suicide in middle-aged men: a prospective cohort study among Korean Vietnam War veterans
Source: PeerJ. 2015 Jul 2;3:e1071. doi: 10.7717/peerj.1071 (PMC4493683; doi:10.7717/peerj.1071)
Supplement: Table S6 — BDI, Beck Depression Inventory; CI, confidence interval; HR, hazard ratio. a. Hazard ratios were calculated using a Cox proportional hazards model over multiple imputed data. b. Classification for drinking status, body mass index, and self-rated health was different from Tables 2 and 3. c. Variables included in the Cox model were age at enrollment, smoking status, drinking status, body mass index, self-rated health, marital status, educational status, household monthly income, participant group, and depressive symptoms (5 categories based on quartiles and the last decile of the total BDI score). d. Sum of the number of suicides may not equal the total suicides (n = 41) due to rounding of averages over multiple imputed data. e. Categories of risk factors were analyzed as ordinal variables. f. 1,300 Korean Won was about one US dollar as of June, 2001. g. Cut-off score based on quartiles and the last decile. [file peerj-03-1071-s006.pdf]

Table S6. Hazard ratios of suicides by depressive symptoms and risk factors in Korean middle-aged men (n=10,238)[a] (Sensitivity analysis)[b]

Categories of risk factors		No. of suicides[d]	Unadjusted analysis		Adjusted analysis[c]	
			p-value	HR (95% CI)	p-value	HR (95% CI)
Age at enrollment	One year increase in age	41	0.24	0.9 (0.9-1.0)	0.30	0.9 (0.8-1.1)
Smoking status	Never smoker	9		1.0 [Reference]		1.0 [Reference]
	Past smoker	7	0.95	1.0 (0.4-2.6)	0.69	0.8 (0.3-2.2)
	Current smoker	25	0.15	1.8 (0.8-3.8)	0.40	1.4 (0.6-3.1)
	p for trend[e]	41	0.11	1.4 (0.9-2.0)	0.32	1.2 (0.8-1.8)
Drinking status	Non-drinker	18		1.0 [Reference]		1.0 [Reference]
	Current drinker	23	0.45	0.8 (0.4-1.5)	0.96	1.0 (0.5-2.0)
Body mass index	Below 23.0	24		1.0 [Reference]		1.0 [Reference]
(kg/m^2)	23.0-24.9	10	0.18	0.6 (0.3-1.3)	0.59	0.8 (0.4-1.7)
	25 or above	7	0.07	0. (0.2-1.0)	0.26	0.6 (0.3-1.4)
	p for trend[e]	41	**0.04**	0.7 (0.4-1.0)	0.25	0.8 (0.5-1.2)
Self-rated health	Very good, good, or fair	10		1.0 [Reference]		1.0 [Reference]
	Poor	16	0.24	1.6 (0.7-3.6)	0.70	0.8 (0.3-2.1)
	Very poor	15	**<0.001**	5.8 (1.8-13.0)	0.21	1.9 (0.7-5.3)
	p for trend[e]	41	**<0.001**	2.5 (1.6-3.9)	0.15	1.5 (0.9-2.6)
Marital status	Living with spouse	35		1.0 [Reference]		1.0 [Reference]
	Living without spouse	6	0.09	2.1 (0.9-5.0)	0.73	1.2 (0.5-2.9)
Educational status	Elementary school or below	16	**0.002**	3.6 (1.6-8.2)	**0.045**	2.4 (1.0-5.5)
	Middle school	15	**0.02**	2.8 (1.2-6.3)	0.09	2.1 (0.9-4.9)

Categories of risk factors		No. of suicides[d]	Unadjusted analysis p-value	Unadjusted analysis HR (95% CI)	Adjusted analysis[c] p-value	Adjusted analysis[c] HR (95% CI)
	High school or above	10		1.0 [Reference]		1.0 [Reference]
	p for trend[e]	41	**<0.001**	0.6 (0.4-0.8)	**0.03**	0.7 (0.5-1.0)
Household income per month (Korean Won)[f]	Below 1,000,000	21	**0.03**	3.7 (1.1-12.2)	0.78	1.2 (0.3-4.5)
	1,000,000-1,990,000	16	0.29	2.0 (0.6-6.8)	0.79	1.2 (0.3-4.4)
	2,000,000 or more	4		1.0 [Reference]		1.0 [Reference]
	p for trend[e]	41	**0.01**	0.5 (0.3-0.9)	0.85	0.9 (0.5-1.7)
Participant group	Health exam group	7	0.46	0.7 (0.3-1.7)	0.77	0.9 (0.4-2.1)
	Survey-only group	34		1.0 [Reference]		1.0 [Reference]
Depressive symptoms[g]	No (0-13)	3		1.0 [Reference]		1.0 [Reference]
	Mild (14-21)	6	0.51	1.6 (0.4-6.8)	0.66	1.4 (0.3-6.0)
	Moderate (22-30)	5	0.70	1.4 (0.3-6.3)	0.99	1.0 (0.2-5.1)
	Severe (31-39)	12	**0.009**	5.4 (1.5-19.4)	0.10	3.4 (0.8-14.4)
	Extreme (40-63)	15	**<0.001**	10.2 (3.0-35.0)	**0.04**	4.8 (1.1-21.8)
	p for trend[e]	41	**<0.001**	1.9 (1.5-2.5)	**0.009**	1.6 (1.1-2.2)

BDI, Beck Depression Inventory; CI, confidence interval; HR, hazard ratio.

a. Hazard ratios were calculated using a Cox proportional hazards model over multiple imputed data

b. Classification for drinking status, body mass index, and self-rated health was different from Tables 2-3.

c. Variables included in the Cox model were age at enrollment, smoking status, drinking status, body mass index, self-rated health, marital status, educational status, household monthly income, participant group, and depressive symptoms (5 categories based on quartiles and the last decile of the total BDI score).

d. Sum of the number of suicides may not equal the total suicides (n=41) due to rounding of averages over multiple imputed data.

e. Categories of risk factors were analyzed as ordinal variables.

f. 1300 Korean Won was about one US dollar as of June, 2001

g. Cut-off score based on quartiles and the last decile
