# Supplementary figures and images for: Depressive symptoms and other risk factors predicting suicide in middle-aged men: a prospective cohort study among Korean Vietnam War veterans
Source: PeerJ. 2015 Jul 2;3:e1071. doi: 10.7717/peerj.1071 (PMC4493683; doi:10.7717/peerj.1071)

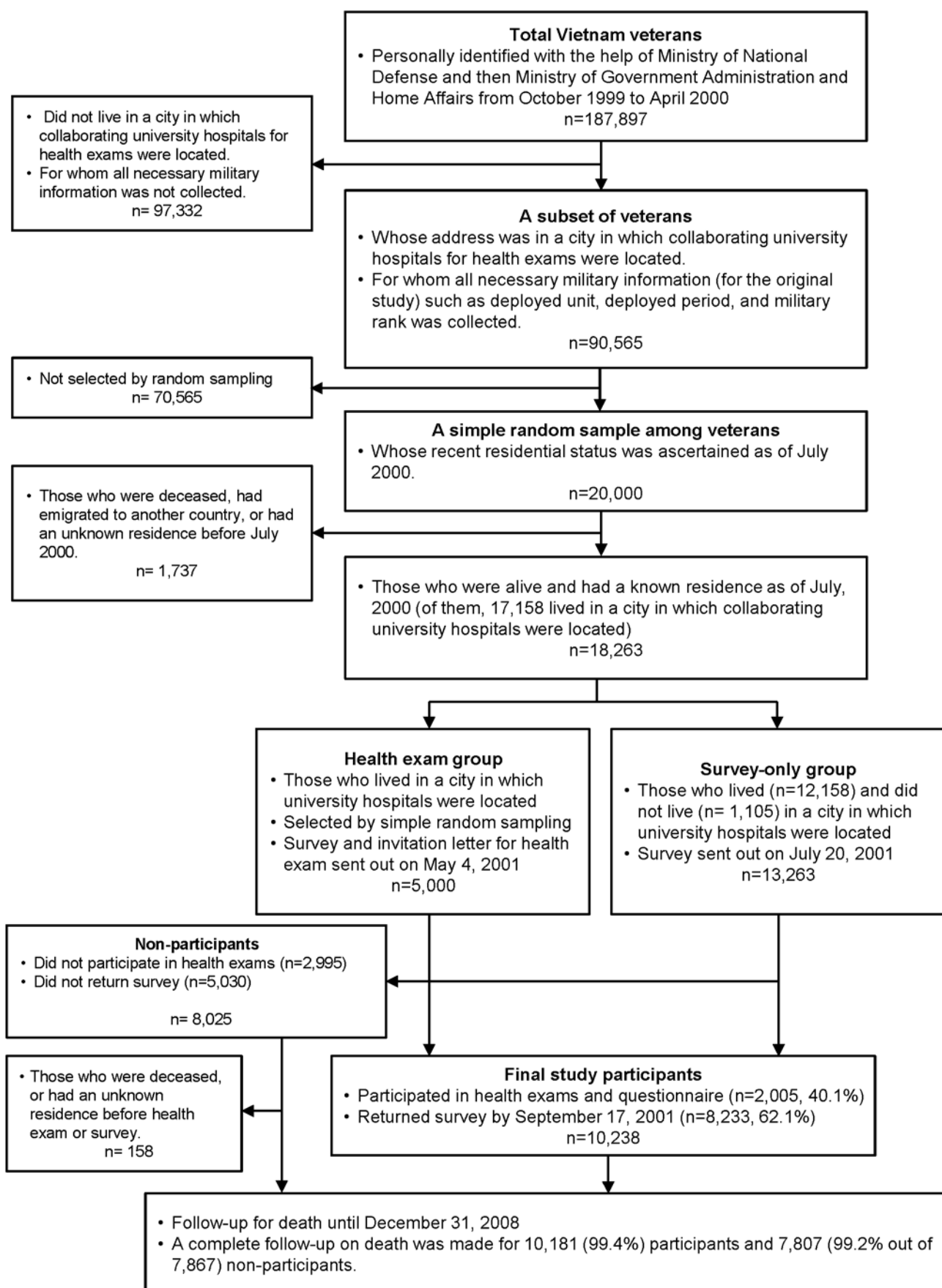

Figure S1. Flow of the study participants

Supplement: Figure S1 [file peerj-03-1071-s007.pdf]
