# Supplementary material for: Depressive symptoms and other risk factors predicting suicide in middle-aged men: a prospective cohort study among Korean Vietnam War veterans
Source: PeerJ. 2015 Jul 2;3:e1071. doi: 10.7717/peerj.1071 (PMC4493683; doi:10.7717/peerj.1071)
Supplement: Figure S2 — Abbreviations: AUC, area under the receiver operating characteristics curve; ROC, receiver operating characteristics. Model 1, unbinned total score. Model 2, cut-off score of 31 or above. [file peerj-03-1071-s008.pdf]

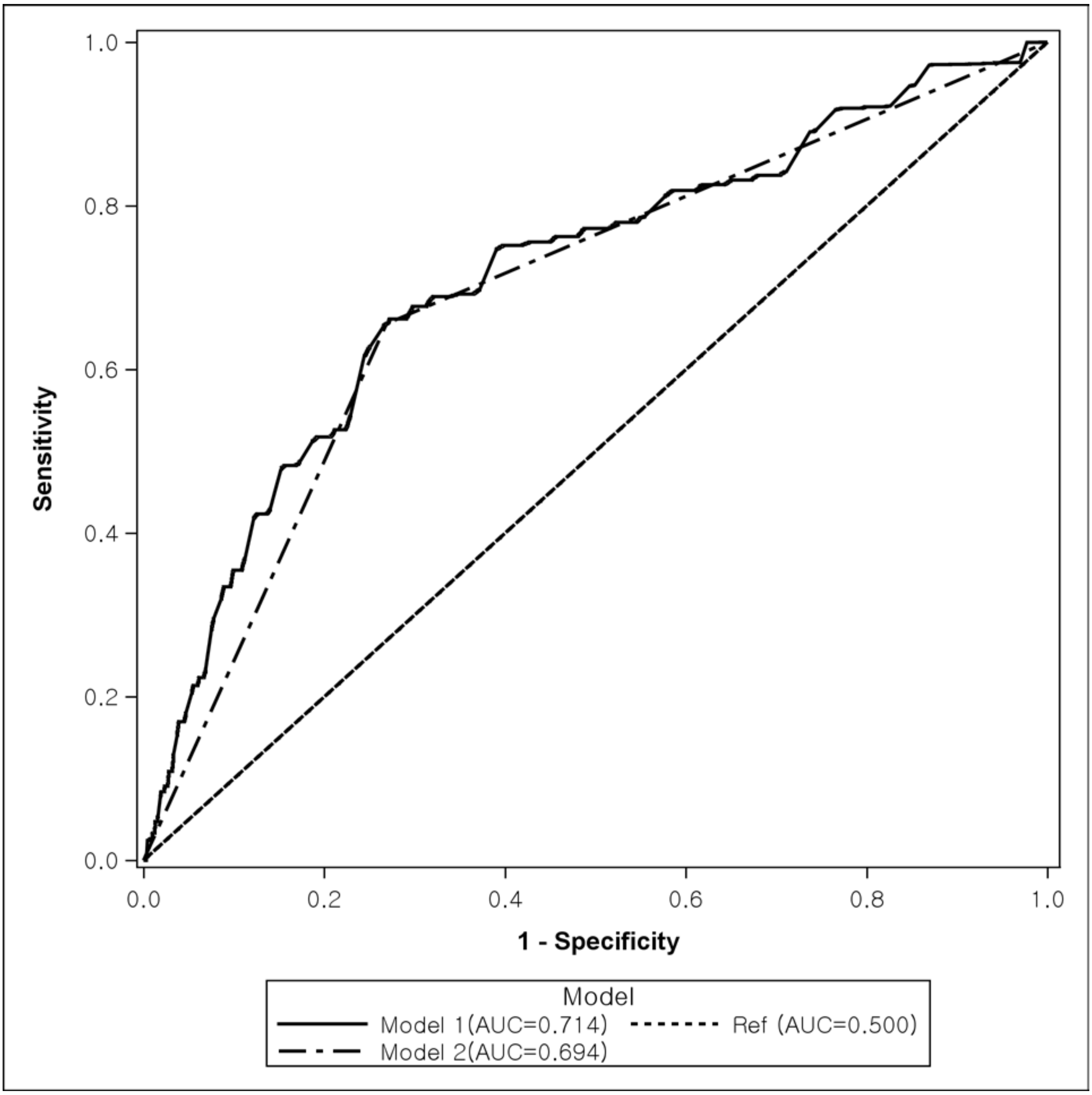

Figure S2. ROC curves of the Beck Depression Inventory (BDI) for suicide death

Abbreviations: AUC, area under the receiver operating characteristics curve; ROC, receiver operating characteristics

Model 1, unbinned total score

Model 2, cut-off score of 31 or above
